# Supplementary material for: Specific ablation of PDGFRβ-overexpressing pericytes with antibody-drug conjugate potently inhibits pathologic ocular neovascularization in mouse models
Source: Commun Med (Lond). 2021 Dec 8;1:58. doi: 10.1038/s43856-021-00059-3 (PMC9053257; doi:10.1038/s43856-021-00059-3)
Supplement: Supplementary file 2 — Supporting information (SI appendix) [file 43856_2021_59_MOESM2_ESM.pdf]

Supplementary Information for

Specific ablation of PDGFRβ-overexpressing pericytes with antibody-drug conjugate potently inhibits pathologic ocular neovascularization in mouse models

Seok Jae Lee[1,2*], Soohyun Kim[3,4,5*,+], Dong Hyun Jo[6], Chang Sik Cho[1], Su Ree Kim[7], Dongmin Kang[7], Jisu Chae[3,4],Duck Kyun Yoo[2,3,5], Suji Ha[3,4], Junho Chung[2,3,4,5‡], Jeong Hun Kim[1,2,8,9‡]

[1]Fight against Angiogenesis-Related Blindness (FARB) Laboratory, Clinical Research Institute, Seoul National University Hospital, Seoul, Republic of Korea; [2]Department of Biomedical Sciences, Seoul National University College of Medicine, Seoul, Republic of Korea; [3]Department of Biochemistry and Molecular Biology, Seoul National University College of Medicine, Seoul, Republic of Korea; [4]Cancer Research Institute, Seoul National University College of Medicine, Seoul, Republic of Korea; [5]Transplantation Research Institute, Seoul National University College of Medicine, Seoul, Republic of Korea; [6]Department of Anatomy & Cell Biology, Seoul National University College of Medicine, Seoul, Republic of Korea; [7]Department of Life Science, Fluorescence Core Imaging Center, Ewha Womans University, Seoul, Republic of Korea; [8]Department of Ophthalmology, Seoul National University College of Medicine, Seoul, Republic of Korea; [9]Advanced Biomedical Research Center, Korea Research Institute of Bioscience & Biotechnology, Daejeon, Republic of Korea

Corresponding authors:

Junho Chung, MD PhD: Department of Biochemistry and Molecular Biology, Seoul National University College of Medicine, Seoul, 03080, Republic of Korea, E-mail: jjhchung@snu.ac.kr

Jeong Hun Kim, MD PhD: Fight against Angiogenesis-Related Blindness (FARB) Laboratory, Biomedical Research Institute, Seoul National University Hospital, and Department of Ophthalmology, Seoul National University College of Medicine, Seoul, 03080, Republic of Korea, E-mail: steph25@snu.ac.kr

[*]These authors contributed equally to this work

[+]Current address: Department of Biochemistry, Stanford University School of Medicine, Stanford, CA 94305, USA; Stanford ChEM-H, Stanford University, Stanford, CA94305

‡Corresponding author. E-mail: jjhchung@snu.ac.kr (JC) or steph25@snu.ac.kr (JHK)

Figure S1. Comparison of the expression level of PDGFRβ in MBVP and NIH3T3.

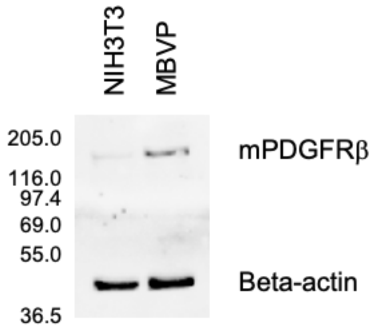

Western blot analysis for mPDGFRβ was performed on lysates obtained from MBVP and NIH3T3 cells. The expression level of mPDGFβ in MBVP cells was higher than that in NIH3T3 cells.

Figure S2. Raw data for comparison of the expression level of PDGFRβ in MBVP and NIH3T3.

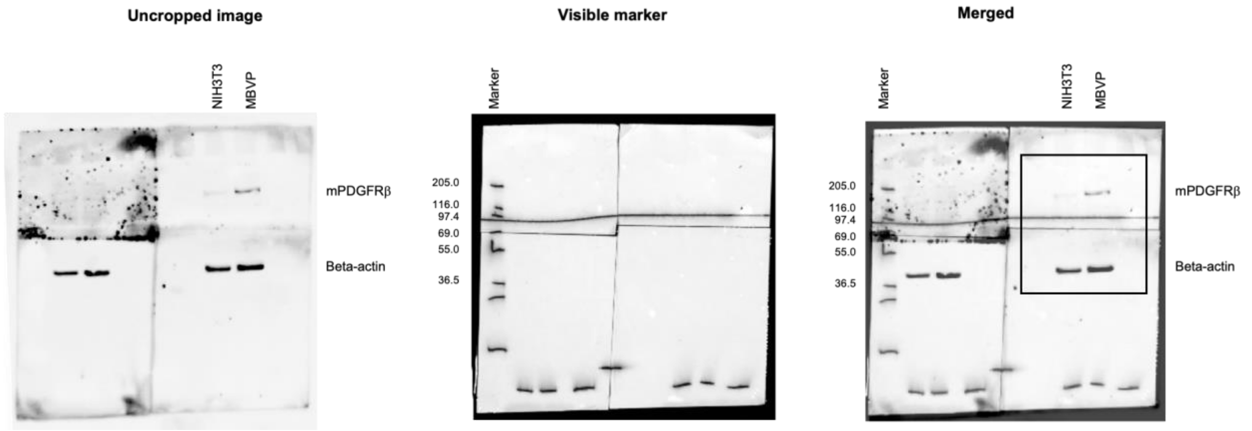

Western blot analysis for mPDGFRβ was performed on lysates obtained from MBVP and NIH3T3 cells. Uncropped image shows bands under chemiluminescence. Visible marker image shows molecular weight marker imaged under visible light. The uncropped image and visible marker were then merged. The region within the black box in the merged image was then cropped and used as Figure S1.

Figure S3. ADC targeting mPDGFRβ and bevacizumab treatment reduce retinal neovascularization in the OIR model.

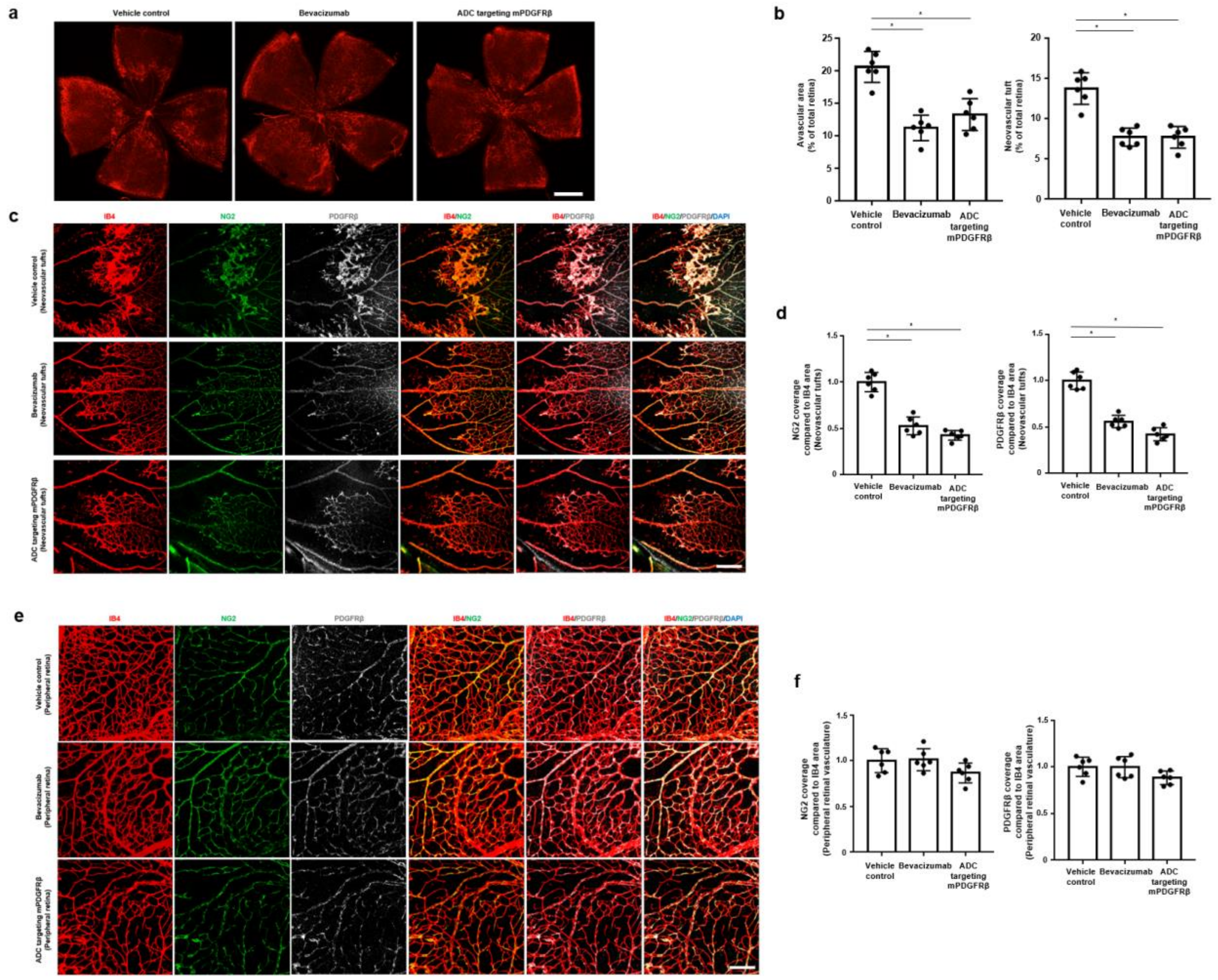

(a) Representative immunofluorescence images of whole-mounted retina samples from OIR pups stained with IB4 (red) to visualize the retinal vessels. OIR-induced pups were treated with vehicle control (PBS), bevacizumab (1 μg/eye, bevacizumab, Avastin®; Genentech Inc., San Francisco, CA), or ADC targeting mPDGFRβ (66.77 pg/eye) through intravitreal injection at P14. Three days after treatment, the mouse eyeballs were dissected to prepare whole-mounted retina samples. Scale bar, 500 μm. (b) Quantification of the avascular area and neovascular tuft area. The avascular area and neovascular tuft area were quantified and presented as percentages of the total area of the retina. All data were analyzed using NIH ImageJ software, and values are presented as percentages of the mean ± SEM (n=6 mice for each group set). **P*<0.01, obtained using one-way ANOVA and Tukey's post-hoc tests. (c) Representative higher-magnification images of the mid-peripheral

region (pathological vascular tuft area) staining with IB4 (red), NG2 (green), and PDGFRβ (gray). Scale bar, 200 μm. (d) Quantification of NG2 (left) and PDGFRβ (right) coverage of IB4+ vessels and values are presented as the mean ± SEM (n=6 mice for each data set). *$P<0.01$, obtained using one-way ANOVA and Tukey's post-hoc tests. (e) Representative higher-magnification images of peripheral region (peripheral vascularized area) staining with IB4, NG2, and PDGFRβ. Scale bar, 200 μm. (f) Quantification of NG2 (left) and PDGFRβ (right) coverage of IB4+ vessels. Error bars represent SEM (n=6 mice for each group set). Source data are provided as a Supplementary Data file.

Figure S4. The effect of ADC targeting mPDGFRβ and bevacizumab on laser-induced CNV.

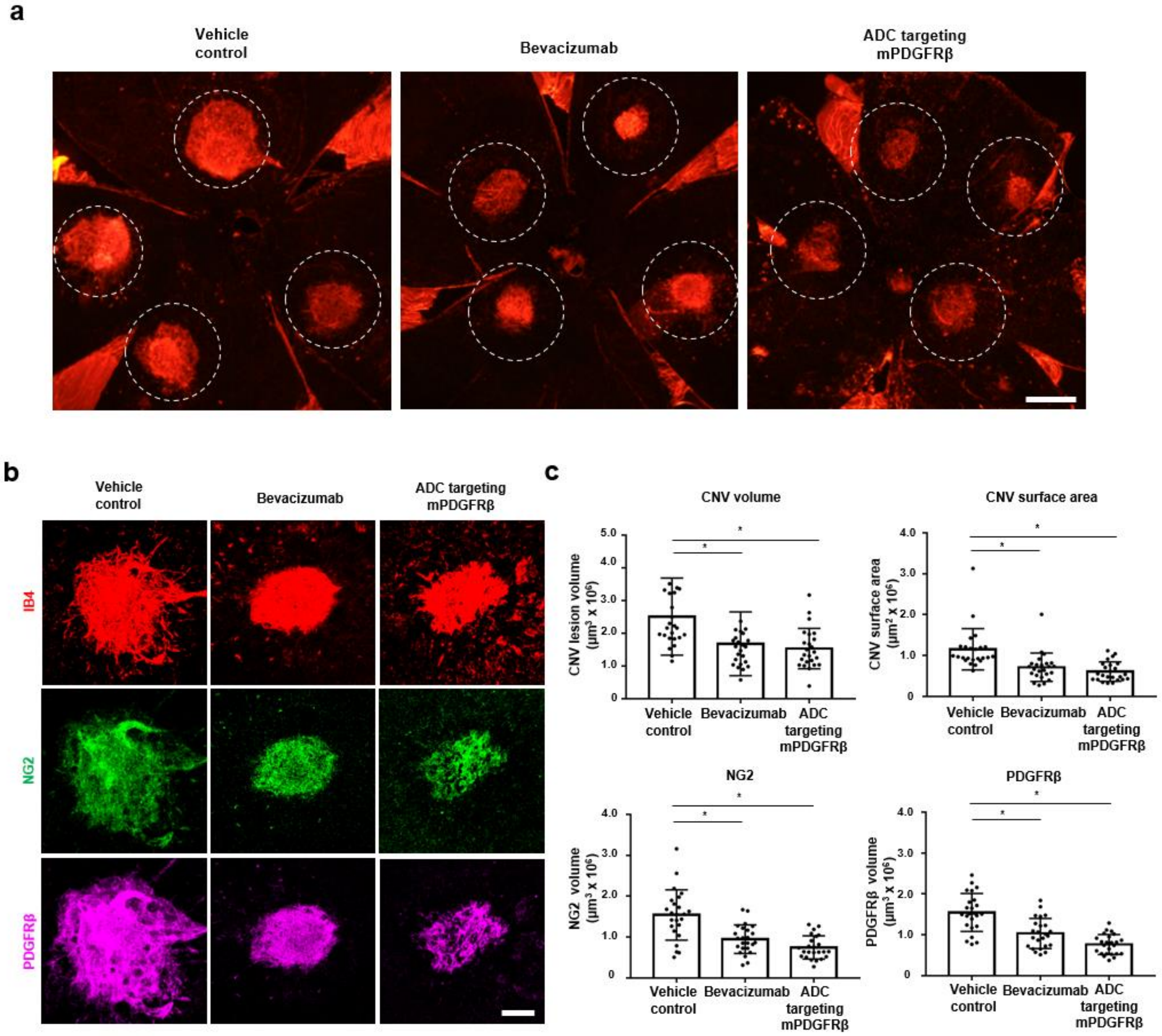

(a) Representative immunofluorescence images of whole-mounted RCS complex staining with IB4 to visualize CNV at 10 days after laser photocoagulation. Six-week-old wild-type C57BL/6J male mice received laser photocoagulation and were treated with vehicle control (PBS), bevacizumab (1 μg/eye, bevacizumab, Avastin®; Genentech Inc., San Francisco, CA), or ADC targeting mPDGFRβ (667.7 pg/eye) through intravitreal injection at 7 days. Three days after treatment, the mouse eyeballs were dissected, and RCS complexes were whole mounted. Scale bar, 500 μm. (b) Representative higher-magnification images of CNV at 10 days after laser photocoagulation and immunostaining with IB4 (red), NG2 (green), and PDGFRβ (pink). Scale bar, 200 μm. (c) Quantitation of CNV volume, CNV area, NG2 volume, and PDGFRβ volume. All

quantitative data were measured using the built-in tools, the LAS X systems (Leica Microsystems, Wetzlar, Germany). Each value represents the mean ± SEM (n=6 mice for each group set). *$P<0.01$, obtained using one-way ANOVA and Tukey's post-hoc tests. Source data are provided as a Supplementary Data file.

Figure S5. Evaluation of blood-retinal barrier leakage and pericyte loss in mice receiving ADC targeting mPDGFRβ.

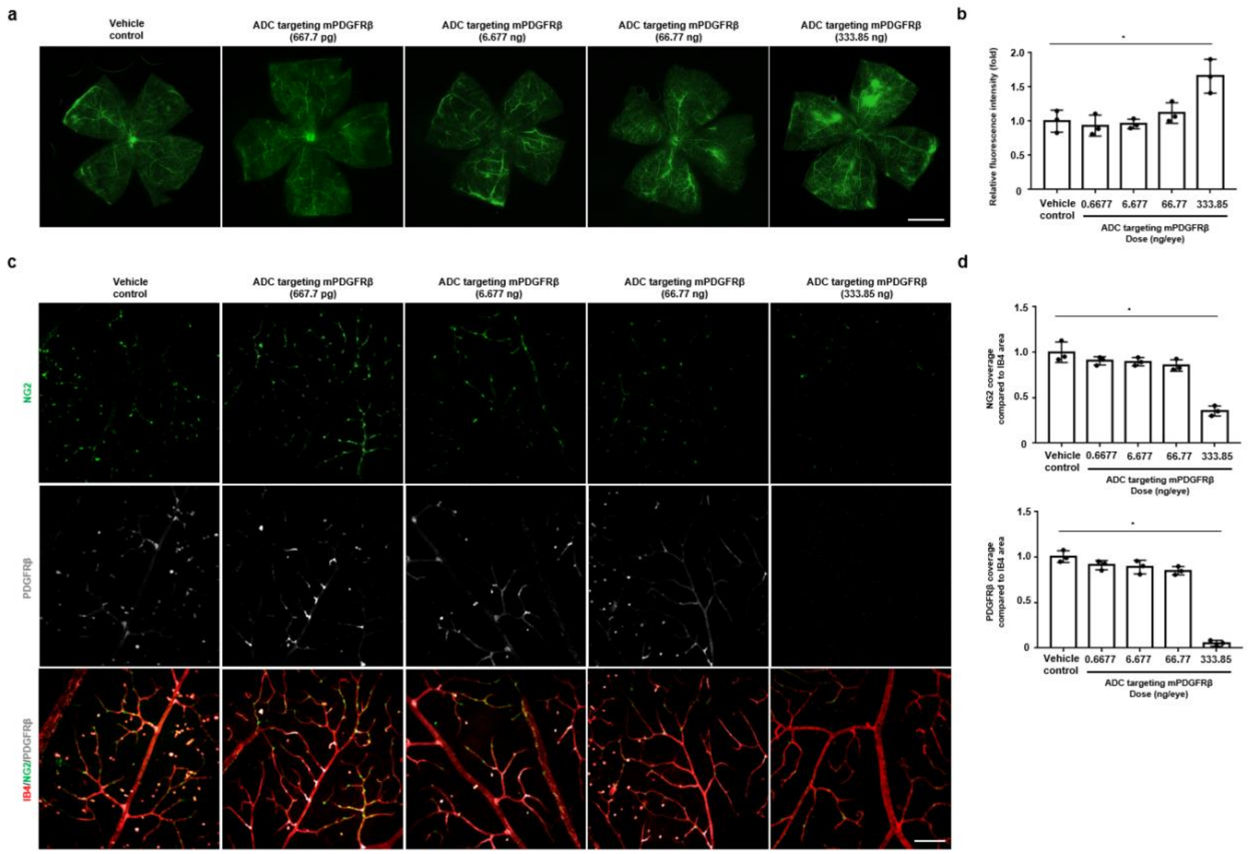

(a) Representative fluorescence images of whole-mounted retinas comparing FITC-dextran (60 kDa, 100 mg/kg) staining pattern as measurement of inner blood-retinal barrier leakage at 7 days after intravitreal injection of vehicle control (PBS) or different doses of ADC targeting mPDGFRβ (667.7 pg ~ 333.85 ng per eye)**.** Scale bar, 500 μm. (b) Quantification of retinal vessel permeability by measuring the fluorescence intensities of FITC-dextran in the whole-mounted retina samples. Each value represents the mean ± SEM (n=3 mice for each group set). *P<0.01, obtained using one-way ANOVA and Tukey’s post-hoc tests. (c) Representative immunofluorescence images of whole-mounted retina samples stained with IB4 (red), NG2 (green), and PDGFRβ (gray) to visualize the retinal vessels and pericytes. Scale bar, 100 μm. (d) Quantification of NG2 (top) and PDGFRβ (bottom) coverage of IB4+ vessels and values are presented as percentages of the mean ± SEM (n=3 mice for each group set). *P<0.01, obtained using one-way ANOVA and Tukey's post-hoc tests. Source data are provided as a Supplementary Data file.

Figure S6. Schematic diagrams depicting the structure of a bispecific scFv-Cκ-scFv antibody and its conjugation process with cotinine-duocarmycin to generate ADCs.

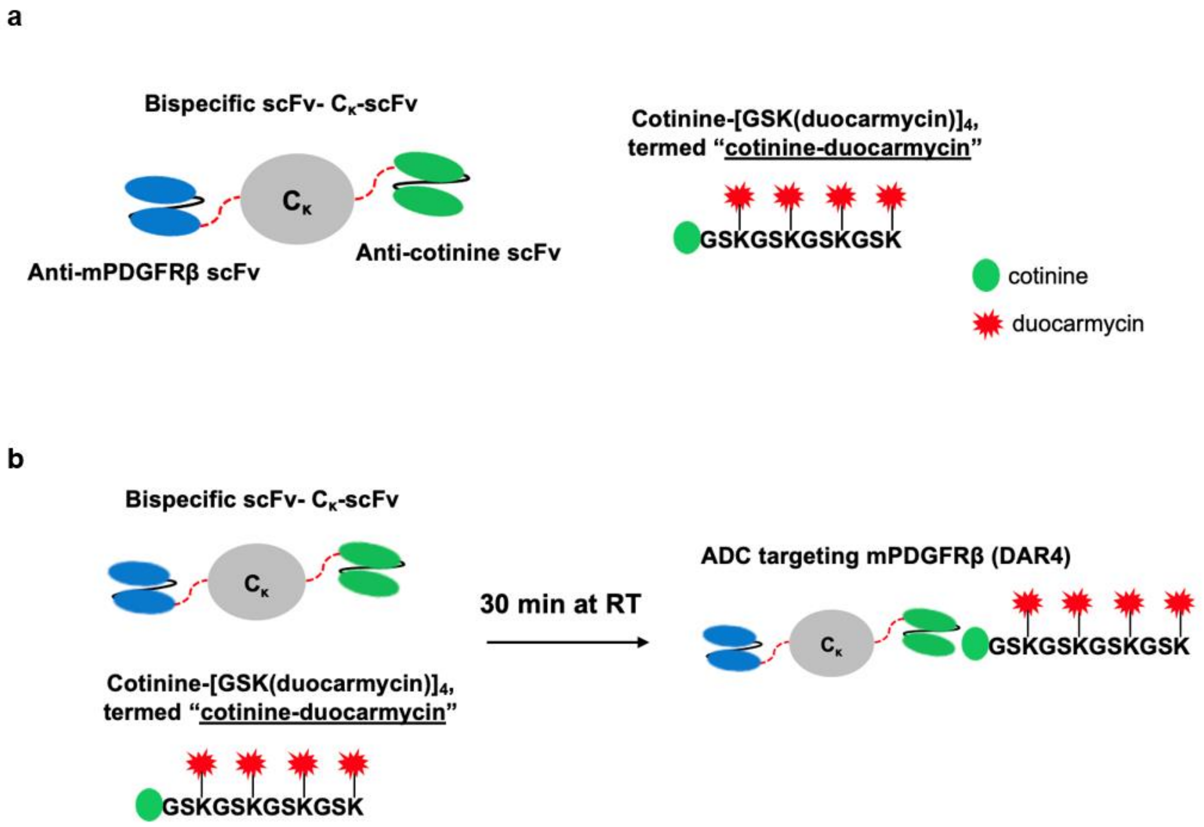

(a) The structures of a bispecific scFv-Cκ-scFv antibody (left) and the drug, cotinine-duocarmycin (right). (b) The schematic representation of the conjugation process of a bispecific scFv-Cκ-scFv antibody and cotinine-duocarmycin. Incubation of the two molecules at room temperature for 30 minutes yields a DAR4 ADC.

Figure S7. LC-MS analysis and RP-HPLC analysis to empirically determine the drug-to-antibody ratio of cotinine-duocarmycin.

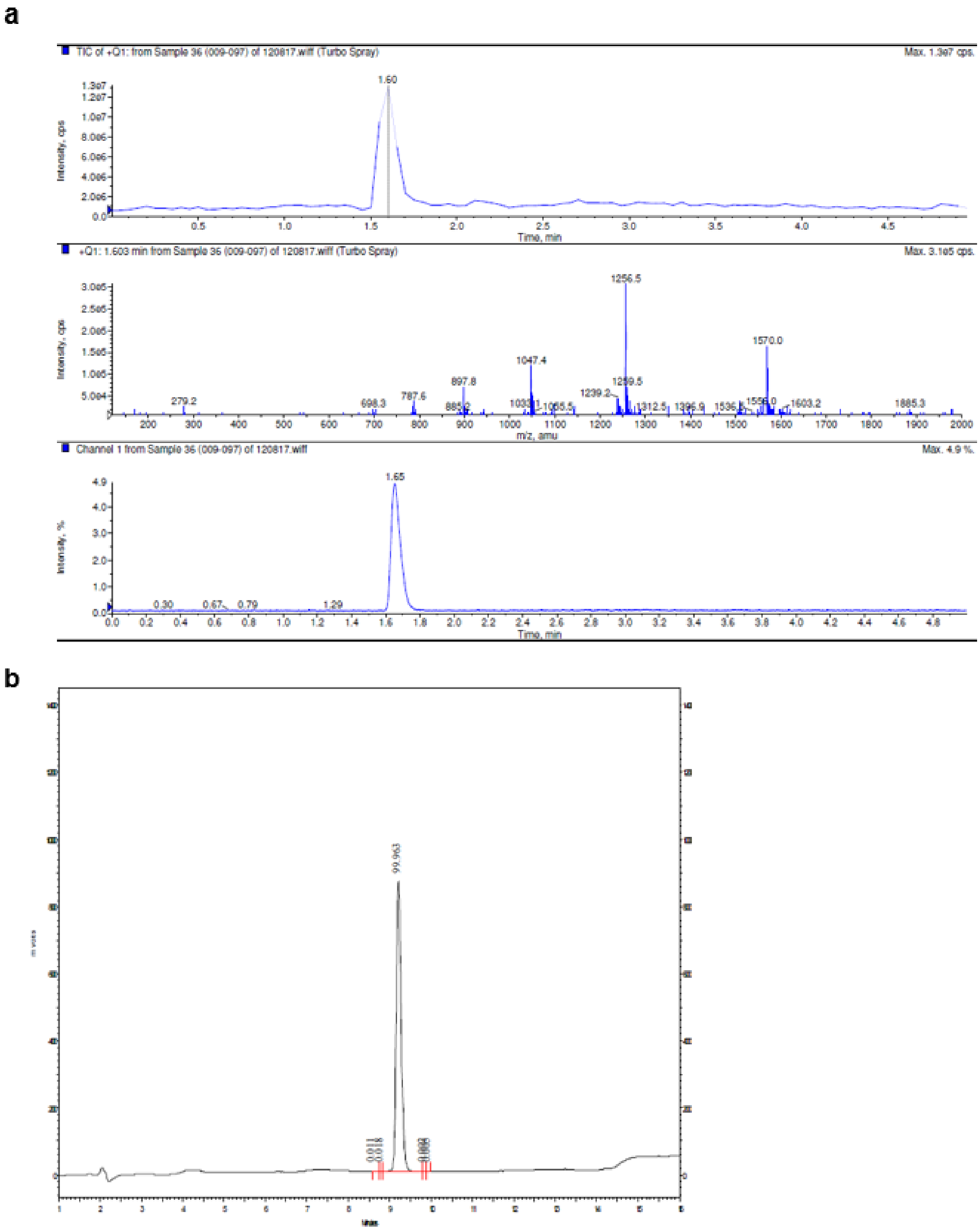

HPLC_Gemini-NX-5 u, C18, 4.6x150 mm
CH3CN-H2O 10 - 70%, 16 min, UV 220 nm
Cot-(GSK-Duocarmycin DM)4_P1712L009097_HPLC

(a) LC-MS analysis. The LC-MS analysis of the final purified conjugate shows a single peak (absorbance at 220 nm) eluting at 1.65 minutes exhibiting an observed parental mass peak of 1570.0 which is consistent with the expected overall mass of the intact molecule (6274) in positive ion mode at a charge (z) of 4. Thus, the mass-to-charge ratio (m/z) of 1570.0 is equivalent to the expected conjugate mass + $4H^+/4$. The resulting LC-MS fragmentation pattern is likewise consistent with this observed parental mass. (b) RP-HPLC analysis. The RP-HPLC analysis under alternative conditions likewise shows a single peak at >99% purity according to peak area integration at 220 nm absorbance. No other additional species (peaks) indicating DAR < 4 or DAR > 4 are detected by LC-MS or alternative RP-HPLC.

Table S1. Cytotoxicity of ADC on mPDGFRβ-expressing MBVP cells

ADC	IC50 (nM)	95% CI
**Anti-mPDGFRβ**		
without mPDGF-BB (-)	0.19	0.14-0.27
with mPDGF-BB (+)	0.30	0.26-0.35
**Control**		
without mPDGF-BB (-)	1.25	0.81-1.92
with mPDGF-BB (+)	1.37	0.90-2.10
**Vehicle control**		
without mPDGF-BB (-)	1.12	0.79-1.59
with mPDGF-BB (+)	1.37	0.90-2.07

Table S2. Body weight of C57BL/6J mice before and after intravitreal treatment of ADC targeting mPDGFRβ

Group	N	Body weight (g)	
		Before treatment	After treatment
Wild-type C57BL/6J (Vehicle control: PBS)	6	23.03 ± 1.87	24.43 ± 1.43
Wild-type C57BL/6J (Control ADC dose: 667.7 pg)	6	22.78 ± 2.11	24.27 ± 0.98
Wild-type C57BL/6J (ADC targeting mPDGFRβ dose: 667.7 pg)	6	22.43 ± 1.41	24.26 ± 1.25
Wild-type C57BL/6J (ADC targeting mPDGFRβ dose: 667.7 pg)	3	23.13 ± 2.21	24.80 ± 2.31
Wild-type C57BL/6J (ADC targeting mPDGFRβ dose: 6.677 ng)	3	22.67 ± 2.01	25.07 ± 1.89
Wild-type C57BL/6J (ADC targeting mPDGFRβ dose: 66.77 ng)	3	22.69 ± 1.37	24.26 ± 0.81
Wild-type C57BL/6J (ADC targeting mPDGFRβ dose: 333.85 ng)	3	23.08 ± 2.08	25.11 ± 1.36

Supplementary Methods

Cell culture

NIH3T3 cells were obtained from the Korean Cell Line Bank (Seoul, Republic of Korea) and maintained in Dulbecco's modified Eagle's medium (DMEM; Welgene, Seoul, Korea) supplemented with 1% penicillin/streptomycin and 10% fetal bovine serum (FBS; GIBCO, Grand Island, NY, USA).

Immunoblotting

MBVP cells and NIH3T3 cells were lysed in RIPA lysis buffer (89900; Thermo Fisher Scientific Inc., Waltham, MA, USA) containing protease inhibitor (5892970001; Sigma-Aldrich, St. Louis, MO, USA) and phosphatase inhibitor (4906845001; Sigma-Aldrich). The protein lysates (3 μg each) were loaded onto NuPage 4-12% Bis-Tris gels (Invitrogen, Carlsbad, CA, USA) following the manufacturer's protocol. The proteins were transferred to nitrocellulose membranes. After blocking the membranes with 5% skim milk (BD Biosciences, Franklin Lakes, NJ, USA) in Tris-buffered saline (TBS) containing 0.2% Tween-20 (TBST) at room temperature for 1 h, the membrane was incubated with rabbit anti-PDGFRβ antibody (C82A3; Cell Signaling Technology, Danvers, MA, USA) diluted 1:1,000 in 5% skim milk in TBST at 4°C overnight. The following day, the membrane was washed in TBST buffer for 10 min four times, and then incubated with peroxidase goat anti-rabbit IgG (111-035-144; Jackson ImmunoResearch Laboratory, West Grove, PA, USA) diluted 1:10,000 in 5% skim milk-TBST at room temperature for 1 h. The membrane was washed in TBST buffer for 10 min four times and subjected to visualization using SuperSignal West Pico PLUS chemiluminescent substrate (34580; Thermo Fisher Scientific Inc.).

Blood-retinal barrier leakage test

To investigate the inner blood-retinal barrier leakage, FITC-dextran (60 kDa, 100 mg/kg) (#FD70S; Sigma-Aldrich, St Louis, MO, USA) was perfused intravenously 1 hour before the sacrifice and retinal whole-mounts were prepared, and blood-retinal barrier leakage was observed under a

fluorescence microscope.
