# Supplementary material for: Specific ablation of PDGFRβ-overexpressing pericytes with antibody-drug conjugate potently inhibits pathologic ocular neovascularization in mouse models
Source: Commun Med (Lond). 2021 Dec 8;1:58. doi: 10.1038/s43856-021-00059-3 (PMC9053257; doi:10.1038/s43856-021-00059-3)
Supplement: Supplementary file 3 — Description of Additional Supplementary Files [file 43856_2021_59_MOESM3_ESM.pdf]

Description of Additional Supplementary Files

File Name: Supplementary Data 1

Description:

Fig. 3c. Avascular area and neovascular tuft of OIR retina
Fig. 3d. NG2 coverage of IB4+ vessels of OIR retina
Fig. 4c. CNV volume, CNV area, NG2 volume, and PDGFRβ volume of laser-induced pathological lesions
Fig. 5a. A/B ratio of H&E staining images and apoptotic cell count of TUNEL staining images
Fig. 5d. Amplitudes of the b-wave by ERG steps
Fig. 5e. Spatial frequency of the OptoMotry test
Supplementary Fig. 3b. Avascular area and neovascular tuft of OIR retina
Supplementary Fig. 3d. NG2 or PDGFRβ coverage of IB4+ vessels of pathological neovascular tuft area
Supplementary Fig. 3f. NG2 or PDGFRβ coverage of IB4+ vessels of peripheral retinal vasculature
Supplementary Fig. 4c. CNV volume, CNV area, NG2 volume, and PDGFRβ volume of laser-induced pathological lesions
Supplementary Fig. 5b. Relative fluorescence intensity of whole-mounted retinas
Supplementary Fig. 5d. NG2 or PDGFRβ coverage of IB4+ vessels of retinal vasculature
